# The Impact of Nonrandom Missingness in Surveillance Data for Population-Level Summaries: Simulation Study

**DOI:** 10.2196/37887

**Published:** 2022-09-09

**Authors:** Paul Samuel Weiss, Lance Allyn Waller

**Affiliations:** 1 Rollins School of Public Health Emory University Atlanta, GA United States

**Keywords:** surveillance, estimation, missing data, population-level estimates, health policy, public health policy, estimates, data, policy decision, bias, response rate

## Abstract

**Background:**

Surveillance data are essential public health resources for guiding policy and allocation of human and capital resources. These data often consist of large collections of information based on nonrandom sample designs. Population estimates based on such data may be impacted by the underlying sample distribution compared to the true population of interest. In this study, we simulate a population of interest and allow response rates to vary in nonrandom ways to illustrate and measure the effect this has on population-based estimates of an important public health policy outcome.

**Objective:**

The aim of this study was to illustrate the effect of nonrandom missingness on population-based survey sample estimation.

**Methods:**

We simulated a population of respondents answering a survey question about their satisfaction with their community’s policy regarding vaccination mandates for government personnel. We allowed response rates to differ between the generally satisfied and dissatisfied and considered the effect of common efforts to control for potential bias such as sampling weights, sample size inflation, and hypothesis tests for determining missingness at random. We compared these conditions via mean squared errors and sampling variability to characterize the bias in estimation arising under these different approaches.

**Results:**

Sample estimates present clear and quantifiable bias, even in the most favorable response profile. On a 5-point Likert scale, nonrandom missingness resulted in errors averaging to almost a full point away from the truth. Efforts to mitigate bias through sample size inflation and sampling weights have negligible effects on the overall results. Additionally, hypothesis testing for departures from random missingness rarely detect the nonrandom missingness across the widest range of response profiles considered.

**Conclusions:**

Our results suggest that assuming surveillance data are missing at random during analysis could provide estimates that are widely different from what we might see in the whole population. Policy decisions based on such potentially biased estimates could be devastating in terms of community disengagement and health disparities. Alternative approaches to analysis that move away from broad generalization of a mismeasured population at risk are necessary to identify the marginalized groups, where overall response may be very different from those observed in measured respondents.

## Introduction

The emergence of COVID-19 in 2019 has given rise to numerous challenges in global health. Many of those challenges have been easily observable and measurable. The intervening months produced countless publications on social distancing and vaccination measures and their resulting effects on the spread of the infection. Even now, epidemiological papers provide current updates on the disease’s differential impact in high-risk populations compared to susceptible people whose risk may not be as high. Most of these analyses were conducted quickly, using available but incomplete data to provide rapid assessments. A challenge that has not been explored in as much detail is how the analysis of incomplete data without proper adjustments may be producing biased results that can lead to detrimental effects as we try to measure knowledge, attitudes, and behaviors related to various aspects of COVID-19.

Public health surveillance data are useful for noninvasively monitoring community health [1]. In some cases, these data are collected as part of an ongoing protocol with defined data elements and quality checks [eg, 11]. Increasingly, however, public health surveillance systems seek to draw conclusions and understanding from a broader collection of data available from administrative, commercial, or other sources [eg, 8-10].

Public health surveillance can be used to address a host of epidemiological questions at a micro level, drilling down to community clusters to identify the who, where, and when of disease concentration. A problem arises when the analyst tries to scale the analysis to the macro level when a nonrandom sample of individuals is used to try to draw inference to a population that the data cannot and do not accurately represent [2-5]. Brick [6] presents a number of potential solutions for reducing nonresponse bias, but these solutions tend to focus on improving response rates as well as statistical adjustment methods for reducing bias in data collections where nonresponse has occurred. In this paper, we quantify and illustrate the range and magnitude of problems encountered when we tried to infer the underlying global properties from an incompletely measured sample where the missingness of the data varied from random to nonrandom. In practice, analysts often turn to sampling weights [6] to control and reduce potential impacts of bias due to nonresponse [2]. In this study, we also examine when and if the use of sampling weights achieves this desired goal in public health surveillance and determine when and if such a strategy makes sense when considering data from a nonrandom microlevel sample for making macrolevel decisions.

Many statistical methods for dealing with missing data require that the data be missing at random (MAR). Investigators turn to methods like those presented in Cohen and Cohen [7], Simonoff [8], or Little and Rubin [9], applying statistical tests to their data to see if they meet this requirement, but these approaches may not provide sufficient rigor for identifying the underlying missingness mechanism, especially if the missingness mechanism is not associated with the auxiliary variables used in the testing [eg, 10]. These approaches are based on a null hypothesis that the data are MAR, and a failure to reject does not provide proof that the null is true. Such approaches also focus on missingness due to the variables involved in the testing and may not have strong statistical power to detect nonrandom missingness due to other reasons [7-9].

An additional approach favored by investigators interested in surveillance involves expanding the sample size through the addition of observations, widening eligibility criteria, or adding additional questions onto an existing large-scale questionnaire [eg, 8,11]. In the case of public-use data sets and surveillance systems, there is often an abundance of observations available for analysis. Extremely large sample sizes are considered to be rich data sources and provide an excellent opportunity to “find something.” Nonprobability samples designed to maximize the number of respondents may present analysts with a wealth of data, but the impact of nonrandom missingness may limit the value of inference drawn from such studies. Although numerous examples of “spam the list” samples and imperfect censuses exist in the literature, we prefer to focus on the statistical impact of such methods rather than calling out our colleagues and peers in this paper for using such methods [eg, 9,11].

Applications of public health surveillance often focus on the data at hand rather than general principles of analytic performance in the presence of nonrandom missingness. In the sections below, we use simulation to explore and illustrate the impact of nonrandom missingness on a single survey item. Our approach allows us to investigate and quantify the error in the estimation of a mean when the randomness of the missingness varies from semicomplete to not complete at all. We also provide an illustration of how increasing the sample size impacts an estimator when the data are not MAR. Finally, we present the results of Cohen and Cohen’s approach [7] for missingness at random for all of our results to assess the performance of this diagnostic approach in identifying when it may be unsafe to assume missingness at random in a given public health surveillance data set. Although it may be well known that, in theory, nonrandom missingness can influence statistical inference, our example provides an illustration of the nature and magnitude of this influence in a simple but realistic setting and in a simple tool for exploration and discovery by readers, students, and researchers.

## Methods

### Overview

A more detailed description of our methods could be seen in Multimedia Appendix 1. Briefly, we present a simulated example of item missingness using a Likert-scale outcome with 5 levels, similar to the kinds of questions often collected in public health surveys. To provide a frame of reference, we consider the outcome to be the answer to the question “how satisfied are you with your community’s efforts to mandate vaccination for local government employees and public servants?” and simulate answers ranging from 1 to 5, one being very dissatisfied and 5 being very satisfied. The simulation uses a discrete random number generator to generate a large (N=100,000) population of potential respondents, where the response pattern is allowed to vary. We present some simulations where an individual’s probability of response is generally uniform across the values, some skewed toward the more satisfied and some skewed toward the less satisfied.

We induce missingness in the data via a uniform random value for each respondent. In our simulation, we compare the effects of data missing completely at random (MCAR) to not missing at random (NMAR) data, where the missingness is not random. We define mechanism as the reason for the data’s missingness, as per the study by Little and Rubin [10]. When the mechanism is completely independent of the survey, then the data are MCAR. When the mechanism is directly associated with the missingness, then the data are NMAR. In the case where the mechanism can be identified and shown to be independent of the data of interest, then the data are MAR. Identifying the mechanism of missingness may be easier to do in the case of item missing data, where nonresponse of certain survey items may be analyzed using completeness in other items. In the case of unit nonresponse, it may be impossible to truly identify the missingness mechanism, as all information on nonrespondents is unavailable. When a mechanism is identified, it may be possible to control for it using multivariable modeling approaches. In this study, we simulate MCAR and NMAR data for a single survey item. For each simulated observation in the population, we also have complete data for race and sex. These demographic items provide auxiliary variables for Cohen and Cohen’s approach [7]. We implement this approach to investigate the test’s ability to effectively detect the NMAR mechanism.

Our simulation replicates 1000 random samples of our overall population and assigns observed values in the sample. Sampling weights [6] are introduced to allow the missing observations to be represented by complete observations.

We quantify the effect of missingness and weighting with the mean squared error (MSE) [11]. The MSE summarizes how far away an estimator is from the truth (on average) and summarizes two components of estimation performance: sampling variability (or sampling error) and bias. A full discussion of the MSE may be found in the Multimedia Appendix 1. Our simulation replicates samples and produces estimator variability, allowing us to estimate sampling variance as a summary of variation in estimation error from sample to sample. The square root of the difference gives us a simulation-based estimate of the estimator’s bias. In the event of rounding leading to negative values of *bias*^2^, we assign the observed bias a value of zero. In our simulation, the bias describes how far away, on average, our sample estimator is away from the true population mean satisfaction, rating in points on a 5-point Likert scale.

We present summary results for the following three population conditions:

- Uniform response across categories (ie, no response is more likely than other).
- Generally satisfied respondents in the population (ie, two satisfied responses are more likely than unsatisfied responses).
- Generally dissatisfied respondents in the population (ie, two dissatisfied responses are more likely than satisfied responses).

Under these conditions, we presented a constant response rate of 90% for the generally satisfied respondents (response of three or higher on the question) and allowed the missingness to vary from 10% to 90% for the dissatisfied respondents to explore the impact of nonrandom missingness. We also compared results for two sample sizes (800 and 8000) to see how this affects the estimators’ behavior. A sample of 800 was chosen for a margin of error of approximately 3.5% for estimating the percentage of those satisfied with the community’s vaccine mandates for government employees and civil servants. The sample size of 8000 was arbitrarily chosen as an inflation by a factor of 10 without specific statistical justification. The simulation was written in SAS 9.4 (Cary, NC). Refer to Multimedia Appendix 2 for the full program.

### Ethical Considerations

No human subjects were involved in this simulation, so no institutional review board approval was necessary.

## Results

### Uniform Response Pattern

We used a uniform response pattern to describe a community without a particularly strong opinion about their government’s efforts toward a vaccine mandate. Our response rates are assigned using a hypothetical convention that people who are generally supportive of public health practices will be inclined to respond to the survey and share their positive opinions, whereas people who are unhappy with the current state of affairs will decline (at a range of levels) to talk about their concerns with a stranger. We hold the response rate constant at 90% for the satisfied members of the community, suggesting their willingness to participate in the survey. We consider scenarios wherein the response rate in the dissatisfied group becomes progressively worse to measure impacts of this differential response on sampling variability, MSE, and bias, in terms of points on a 5-point Likert scale. We also report results after calculating weighted means in an attempt to adjust for nonresponse for samples from this community.

**Figure 1:**
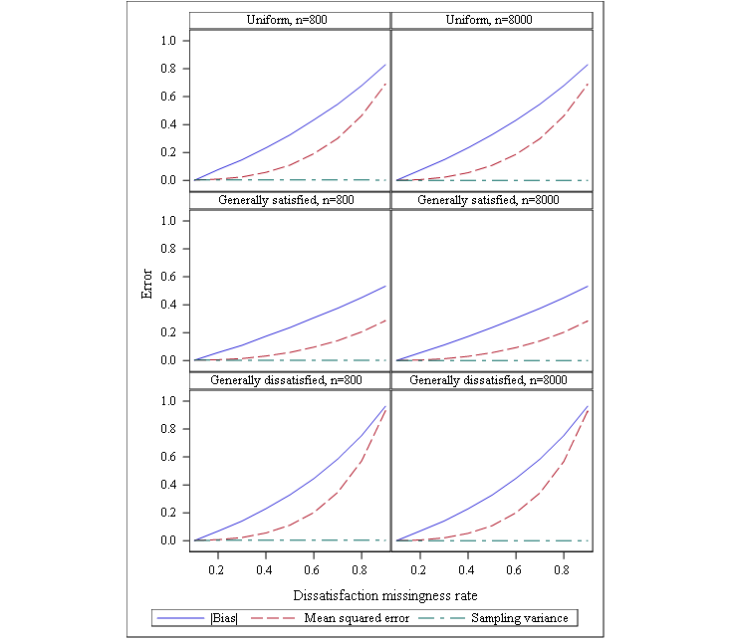
Mean squared error (MSE), sampling variance, and bias by sample size and response pattern.

The first row in Figure 1 compares the performance of the estimator as the nonresponse rate becomes increasingly worse in the dissatisfied group. When response rates are similar between those who are generally satisfied and those who are generally dissatisfied, we see little evidence of bias; as the gap widens in response disparity, we can see a clear upward trend in MSE. Sampling variability appears to be relatively unaffected, but the sharp increase in bias indicates that although our estimator has considerable precision, our intervals are not likely to contain the true satisfaction rating of our full population. At its worst, the estimated policy satisfaction rating is off almost an entire scale point compared to the population truth. The first columns of Table 1 show how often Cohen and Cohen’s approach [7] correctly identifies a departure from missingness at random. We see approximately 5% of the samples presenting with an association between one of the demographic variables and missingness, but we rarely see evidence to indicate missingness not at random using this approach, suggesting low statistical power to detect nonrandom missingness in our setting. In addition, Table 1 also reveals that the bias appears to be considerable even when adjusting for nonrandom missingness using traditional adjustment weights. That indicates the use of sampling weights will not remove the underlying problem.

Interestingly, our results remained consistent when we expanded the sample size (Table 2). Increasing the sample size does not appear to reduce the bias in the estimator nor does it seem to have an impact on its overall variability. The inflated sample size neither reduced nor inflated the inherent bias of the estimator and had no apparent impact on the power of the Cohen and Cohens’ approach to detect departures from missingness at random. Since MSE is a linear combination of variance and *bias^2^*, we see no change in these quantities when the sample size increases. The sampling variance is improving, but negligibly so when compared to the impact the bias has on the quality of the estimator. The missing data contribute to a heavily biased satisfaction estimate, so the MSE or the average distance of our sample estimates from the true mean, is driven by the Bias component. The sample means vary very little between replicates, whereas they vary greatly from the true mean of the population.

**Table 1:** Number of samples out of 1000, where Cohen and Cohen’s approach [7] identifies nonrandom missingness using sex and race based on a sample size of 800.

Dissatisfied nonresponse rate	Uniform, n				Generally satisfied, n				Generally dissatisfied, n		
	Race	Sex	Both	Race		Sex	Both	Race		Sex	Both
10%	50	43	3	41		54	0	49		55	3
20%	46	52	4	52		53	5	59		49	2
30%	62	46	3	55		55	4	52		57	4
40%	45	48	3	54		69	3	57		61	6
50%	42	48	0	52		41	3	37		47	1
60%	51	37	1	43		40	1	46		59	1
70%	55	42	5	46		59	2	56		52	3
80%	53	47	1	50		63	4	51		61	2
90%	49	38	3	70		53	2	57		57	3

**Table 2:** Number of samples out of 1000, where Cohen and Cohen’s approach [7] identifies nonrandom missingness using sex and race based on a sample size of 8000.

Dissatisfied nonresponse rate	Uniform, n				Generally satisfied, n				Generally dissatisfied, n		
	Race	Sex	Both	Race		Sex	Both	Race		Sex	Both
10%	34	38	1	43		44	1	43		52	2
20%	36	50	2	34		37	3	32		39	1
30%	35	43	2	39		37	3	36		43	2
40%	42	40	1	46		43	2	37		52	6
50%	34	49	0	45		58	3	42		51	1
60%	46	43	4	40		57	3	50		37	1
70%	38	50	2	53		44	5	48		36	0
80%	42	29	1	51		43	2	49		50	2
90%	29	31	2	60		60	3	46		51	1

### Generally Satisfied Response Pattern

When the simulated respondents were generally satisfied, we observed less overall missingness in the data, even as the nonresponse rate for dissatisfied respondents increased. The second row in Figure 1 shows the estimator’s behavior under a favorable response profile. In this population, we see that bias is considerably reduced because our sample is more representative of a truly more favorable population. We see that sampling variability is comparable between response profiles because the underlying sampling distribution of the estimator has not changed, so the variation of the estimates from sample to sample is unaffected. However, since these sample estimators are closer to the truth, we also see an arrested increase in the MSE and bias even as the dissatisfied response rate falls. The simulation also reveals that increasing the sample size had little impact on the bias in either direction.

### Generally Dissatisfied Response Pattern

The third row of Figure 1 illustrates a missingness pattern where a large part of the population is both disenfranchised and disinclined to participate in the survey. In this scenario, the respondents present a much different population estimate than what is actually true. As with the other scenarios, there is little sample-to-sample variability. In the generally satisfied population, this posed a different kind of problem, as the respondents who were less likely to respond comprised a smaller part of the population as a whole. In the generally dissatisfied population, however, the respondent-based estimate was far removed from the population’s truth; the resulting naive confidence intervals have no reliable coverage, while providing the appearance of high precision, suggesting a mostly satisfied population, even after adjusting with sampling weights. The estimator becomes biased much more quickly in the generally dissatisfied population, where nonresponse rates of 40%-50% result in the same apparent bias as much higher nonresponse rates in the two other populations considered. As in the previous case, the simulation results reveal that increasing the sample size does not appear to make a significant difference in this effect, and Cohen and Cohens’ approach does not reliably result in a detection of the missingness’ departure from randomness.

## Discussion

### Principal Findings

Our simulations illustrate the impact nonrandom missing data can have on population-based estimates even when analyzing a fairly simple survey sample. What we present in our examples indicate that basic diagnostic tests of missingness at random or the use of sampling weights do not automatically control for such biases and are not simple guarantees or workarounds to improve the quality of estimates.

Statistical discussions of missingness tend to focus on reducing nonresponse in the survey implementation [6] or fixing the data in the analysis [10]. These methods can be elegant and applicable to data collected under a specified design. Under MCAR missingness, a sample is simply reduced but not in a manner that generates bias. Under NMAR missingness, however, the “true” observed sample is a combination of the design (with known probabilities of selection) and the missingness pattern (typically with unknown probability of observation).

In surveillance data, particularly in a public health crisis, where data are needed quickly, existing surveys often are repurposed for additional data collection, or analysts include convenient data of unknown (if any) design. In this repurposed use (eg, through the addition of COVID-19 questions to ongoing surveys), we may well expect new (and unknown) patterns of missingness. Adjusting for design alone (via the design-based weights based on designed probability of selection but not necessarily probability of response) can adapt estimates for the anticipated design; however as seen above, important impacts of new causes of missingness will be missed. Specifically, the examples in our study illustrate how dissonance between sampling weights (adjusting for the probability of “selection”) and patterns of missingness (which changes the probability of “response”) can result in bias. Such impacts can be mitigated if the missingness occurs in subpopulations with low weights (as in our generally satisfied population example) but can be inflated if the missingness occurs in subpopulations receiving high sampling weights (as in our generally dissatisfied population example). Unless we know both the probability of selection and the probability of response, we cannot see the full picture and cannot adjust estimates appropriately with traditional reweighting methods.

As our simple example illustrates, the application of design weights should not be considered as a panacea for the challenges of extending survey designs for surveillance purposes. A closer look at Figure 1 reveals evidence as to why caution is necessary when applying weights in practice, particularly in settings where probabilities of response are unknown. In our simulation examples, while the MSE and bias trend upward as the dissatisfied response rate decreases, the sampling variability remains constant. The sampling variability is the essential statistic for producing confidence intervals and evaluating hypothesis tests—two broad statistical applications of inferential methods. We can see that confidence intervals produced from surveillance data may have the desired width determined by the sample size calculation, but the bias (due to nonrandom missingness) will result in a precise interval around the wrong number, potentially leading to very poor decisions, policies, and their consequences. Since sampling variability does not fully account for deviation from the truth the way the MSE does, in practice, we may never truly know how far away our sample’s estimate is from the true but unknown population value. If we assume that the missingness is completely at random and produce biased estimates, our reported estimates may (and most likely will) lead to incorrect decisions with potentially long-standing public health implications.

A larger problem comes from the intention to use surveillance data to make global statements about a community. Extrapolation is often mentioned as a concern in modeling but rarely translated to estimators drawn from a nonrandom sample inferring parameters of a larger population. Our simulator shows that as the response rate becomes increasingly worse in a population subgroup, the sample’s effectiveness at representing the larger community abates, in many cases rather drastically. Using data from a sample with unknown probabilities of observation, particularly survey data where the data may not be MAR, is a clear example of extrapolation. Ultimately, a failure to adequately represent a marginalized population may lead to political and social unrest. Policy decisions based on such data could result in creating or widening disparities already detrimental to social justice and health equity outcomes.

The simulations we showed in our study illustrate that estimates from survey samples have the potential to be heavily biased when extended beyond their design, especially in the presence of differential missingness due to imbalanced probability of response. Although the potential of such bias is known in theory, our simulations provide a basic but practical illustration of the potential magnitude of the problem. We note that these simulations represent a simplified (but perhaps not rare) illustration of the problem; the direction and magnitude of the bias can and likely will vary considerably as the relationship missingness shares with the survey changes. We contend that surveys with missing data will rarely (if ever) be random to some extent in surveillance settings and recommend considerable caution when applying survey weights based on sampling plans alone without consideration of potential differential missingness. In particular, we recommend that thoughtful summaries of potential biases accompany analyses and interpretation, especially those drawing from multiple available data sources. We recommend that, rather than using the survey data to look upward to the community, analysts should be encouraged to consider looking down to the observed population, instead.

Although it makes analytical sense to assume the data are MAR, this decision may come with a sizable cost. If we assume missingness at random in error, we reach conclusions that are far from the truth and could lead to devastating social consequences. If we assume that the missingness is not random in error, we make more cautious conclusions and open avenues to better identify and understand potentially underserved sections of our population of interest. Erring on the side of nonrandom missingness leads to a more socially responsible analysis of all the available information.

One limitation of our study is that we applied simple random sampling to simulate the survey experience where most surveillance data sets are multistage cluster designs. We note that a more complex design typically would likely lead to an inflation in the sampling variability but would not reduce the mean squared error or bias inherent in the differential response. In our examples, we also only considered three response patterns and arbitrarily assigned the population responses based on our own characterization of stronger satisfaction and dissatisfaction propensity. Our simulator is available to readers (See Multimedia Appendix 2) and is easily reprogrammed for more complex population response profiles. The simulator can also be modified to measure other kinds of response types (eg, continuous or binary) in addition to our Likert scale example. We limited our analysis to a basic survey design and response because the setting clearly illustrates our point and mirrors a very common setting when analyzing surveillance data.

### Conclusions

Surveillance is an essential component of public health practice. Surveillance data allow us to produce useful descriptive measures to characterize the movement of disease through a population at risk. The current pandemic has produced vast amounts of data, much of which come from nonrandom samples or are drawn from surveys where data missingness patterns can obscure original sampling plans to the point that traditional sampling weights alone cannot provide proper adjustments to estimates. Our examples suggest an opportunity to develop new methods that move away from classical design-only approaches and move toward methods that explore designs for data collection and adjustment for patterns in data completeness that let us use the information more effectively for making better public health decisions for the entire population.

## Appendix

Multimedia Appendix 1 Full description of methods.

Multimedia Appendix 2 SAS Simulation Macro.

## Abbreviations

MSE: mean squared error
MAR: missing at random
MCAR: missing completely at random
NMAR: not missing at random
