# Supplementary material for: The Impact of Nonrandom Missingness in Surveillance Data for Population-Level Summaries: Simulation Study
Source: JMIR Public Health Surveill. 2022 Sep 9;8(9):e37887. doi: 10.2196/37887 (PMC9508670; doi:10.2196/37887)
Supplement: Multimedia Appendix 1 [file publichealth_v8i9e37887_app1.docx]

Methodological Appendix

## METHODS

To mirror many applications but maintain simplicity, we present a simulated example of item missingness using a Likert-scale outcome with five levels, similar to the kinds of questions often collected in public health surveys. To provide a frame of reference, we consider the outcome the answer to the question, “How satisfied are you with your community’s efforts to mandate vaccination for local government employees and public servants?” and simulate answers ranging from one to five, one being very dissatisfied and five being very satisfied. The simulation uses a discrete random number generator to generate a large (*N*=100,000) population of potential respondents where the response pattern is allowed to vary. We present simulations where an individual’s probability of response is generally uniform across the values, skewed towards the more satisfied and skewed towards the less satisfied.

We induce missingness in the data via a uniform random value for each respondent. Data that are missing completely at random (MCAR) have no underlying mechanism driving their missingness [10]. We simulate missingness completely at random in our population by assigning the same non-response rate across the different values of the question responses. Data that are not MCAR could be missing due to an identifiable reason (mechanism) that is not tied to the survey item (missing at random: MAR) or missing due specifically to the item being measured (not missing at random: NMAR). To properly assume MAR it must be possible to identify the mechanism for missingness. This may be easier to do in the case of item missing data, where non-response of certain survey items may be analyzed using completeness in other items. In the case of unit non-response it may be impossible to truly identify the missingness mechanism as all information on non-respondents is unavailable. When a mechanism is identified it may be possible to control for it using multivariable modeling approaches. Our simulation focuses on comparing MCAR to NMAR only although our MCAR results may be comparable to what might be observed in an MAR setting as well if the mechanism is assumed to be identified and not directly responsible for the missingness in the item of interest. We create NMAR data by specifying the missingness rate differentially for different levels of response, where people who are less satisfied respond with a different rate than people who are neutral or more satisfied. For each simulated observation in the population we also have complete data for race and sex. These demographic items provide auxiliary variables for Cohen and Cohen’s test. [7] We implement Cohen and Cohen in an effort to determine if the item missingness may be attributed in some way to these demographic variables.

Our simulation replicates 1,000 random samples of our overall population and assigns observed values in the sample. Under an assumed simple random sample, the standard non-response adjustment applied to survey data is given by an inverse-probability weight based on the inverse of the proportion missing, i.e.

$$w=\frac{n}{n_{obs}}$$

where *n* is the intended sample size and *n_obs_* is the number of measured (non-missing) data values in the sample. We can apply this adjustment to the observed data within the sample where *n_obs_* varies from sample to sample (due to the random missingness mechanism) but the overall *n* is fixed.

We quantify the effect of missingness and weighting with the Mean Squared Error (MSE). The MSE summarizes how far away an estimator is from the truth (on average) and summarizes two components of estimation performance: sampling variability and bias. Random samples will produce estimates that vary around the true parameter of interest, due to different samples taken of the entire population. This sample-to-sample variation is the sampling variability or sampling error. Bias represents a systematic over- or under-estimate of the truth. If an estimator is unbiased then its MSE will be the same as its sampling variability. The difference between an estimator’s sampling variance and MSE provides an estimate of bias via the well-known relationship [11] between these quantities, i.e.:

$$MSE=Variance+{Bias}^{2}$$

In our simulation, we calculate the simulated MSE by subtracting the true population mean from each simulated sample mean, squaring those differences and finding their mean. Replicating samples produces estimator variability and allows us to estimate sampling variance as a summary of sample-to-sample variation. The square root of the difference gives us a simulation-based estimate of the estimator’s bias. In the event of rounding leading to negative values of *Bias*^2^, we assign the observed bias a value of zero.

We present summary results for three population conditions:

1. Uniform response across categories (i.e., no response is more likely than other),
2. Generally satisfied respondents in the population (i.e., the two satisfied responses are more likely than the unsatisfied responses), and
3. Generally dissatisfied respondents in the population (i.e., the two dissatisfied responses are more likely than the satisfied responses).

Under these conditions, we present a constant response rate of 90% for generally satisfied respondents (response of three or higher on the question) and allow the missingness to vary from 10% to 90% for the dissatisfied respondents to explore the impact of non-random missingness. We also compare results for two sample sizes (800, 8,000) to see how this affects the estimators’ behavior. A sample of 800 was chosen for a margin of error of approximately ±3.5% for estimating the percent satisfied with the community’s vaccine mandates for government employees and civil servants. The sample size of 8,000 was arbitrarily chosen as an inflation by a factor of 10 without specific statistical justification.
