# Supplementary material for: The Impact of Nonrandom Missingness in Surveillance Data for Population-Level Summaries: Simulation Study
Source: JMIR Public Health Surveill. 2022 Sep 9;8(9):e37887. doi: 10.2196/37887 (PMC9508670; doi:10.2196/37887)
Supplement: Multimedia Appendix 2 [file publichealth_v8i9e37887_app2.docx]

*** Submit macro first ***;

options mprint mlogic symbolgen;

**%Macro** wtsim (seed,

numresp, resp,

mr_sat, mr_dissat, nrcut,

n, popsz, numsamples,

popstats, row, nmartest);

data work.pop;

*** Simulate a Likert-scale response (5-point) where 1 indicates

great dissatisfaction and 5 indicates great satisfaction.

User specifies the response pattern as proportions that sum to 1

***;

array p (&numresp) p1-p&numresp (&resp);

do i = **1** to &popsz;

y = rantbl(&seed,of p1-p&numresp); *** True responses to item ***;

*** Introduce missingness ***;

mr = ranuni(**0**); *** Generate a uniform value from zero to one ***;

if y lt &nrcut then do;

if mr lt &mr_dissat then y_obs = **.**; ** Create missing data **;

else y_obs=y;

racegr = rantbl(**0**,**0.7**,**0.2**,**0.1**); ** Adjust race percentages (W/B/O) **;

sex_bin = rantbl(**0**,**0.5**,**0.5**); ** Adjust sex percentages (M/F) **;

end; else

if y ge &nrcut then do;

if mr lt &mr_sat then y_obs = **.**; ** Create missing data **;

else y_obs=y;

racegr = rantbl(**0**,**0.7**,**0.2**,**0.1**); ** Adjust race percentages (W/B/O) **;

sex_bin = rantbl(**0**,**0.5**,**0.5**); ** Adjust sex percentages (M/F) **;

end;

output;

end;

drop p1-p&numresp i mr; ** Clean up dataset **;

label y="True respondent value"

y_obs="Observed respondent value"

;

run;

*** Generate population parameter information ***;

%if &popstats=T %then %do;

proc means data=work.pop;

var y y_obs;

title1 "True and Observable Population Means";

title2 "Satisfaction Missing Rate: &mr_sat";

title3 "Dissatisfaction Missing Rate: &mr_dissat";

run;

%end;

proc means noprint data=work.pop; *** Extract true population response mean ***;

var y;

output out=work.truth mean=ybar;

run;

data _null_;

set work.truth; *** Create a macro variable for the true population mean ***;

call symput ("true_mean",ybar);

run;

proc surveyselect data=work.pop out=work.samples n=&n

noprint method=srs rep=&numsamples;

run;

proc means noprint data=work.samples;

var y_obs;

by replicate;

output out=work.means n=n_obs; *** Get the valid n for calculating weights ***;

run;

data work.survey;

merge work.samples work.means;

by replicate;

nrweight = &n/n_obs; *** Blanket non-response adjustment ***;

ymiss = (y_obs = **.**); ** Creates an indicator of y missing (1) or not (0);

** This indicator will be used for demographic testing

of missingness

**;

run;

proc means noprint data=work.survey;

var y_obs;

weight nrweight;

by replicate;

output out=work.results1 mean=ybar;

run;

%if &nmartest=**1** %then %do;

*** Using Sex and Race to test for NMAR ***;

ods select none;

proc freq data=work.survey;

by replicate;

tables racegr*ymiss / chisq ;

ods output chisq=work.racetest;

run;

proc sort data=work.racetest;

by replicate statistic;

run;

proc freq data=work.survey;

by replicate;

tables sex_bin*ymiss / chisq ;

ods output chisq=work.sextest;

run;

proc sort data=work.sextest;

by replicate statistic;

run;

ods select all;

data work.results2;

merge work.racetest(rename=(Prob=Race_pval)) work.sextest (rename=(Prob=Sex_pval));

by replicate statistic;

if statistic="Chi-Square";

race_reject=(Race_Pval le **0.05**);

sex_reject=(Sex_Pval le **0.05**);

drate = &mr_dissat;

srate = &mr_sat;

conclude_nmar = (race_reject = **1** AND sex_reject = **1**);

label drate = "Dissatisfied Non-response Rate"

srate = "Satisfied Non-response Rate"

race_reject = "Declare Missing and Race are associated"

sex_reject = "Declare Missing and Sex are associated"

conclude_nmar = "Declare data are NMAR due to demographic association"

;

run;

proc means noprint data=work.results2;

var drate srate race_reject sex_reject conclude_nmar;

output out=work.nmartest&row mean= drate srate

race_reject sex_reject conclude_nmar;

run;

proc append base=work.test data=work.nmartest&row;

run;

%end;

data work.mse;

set work.results1;

truth = &true_mean; *** Taken from the original population -- the true mean y ***;

error_sq = (ybar - truth)****2**;

run;

proc means data=work.mse noprint;

var ybar error_sq;

title1 "Mean Squared Error of Sample Means compared to Truth";

title2 "Compared to Sampling Variability of Y-bar (Var(ybar))";

title3 "Satisfaction Missing Rate: &mr_sat";

title4 "Dissatisfaction Missing Rate: &mr_dissat";

output out=work.save var= sampvar a

mean=b mse

;

run;

%if &row ne **.** %then %do;

data work.row&row;

label drate="Dissatisfaction Missingness Rate"

srate="Satisfaction Missingness Rate"

MSE="Mean Squared Error"

sampvar="Sampling Variance"

bias="|Bias|"

;

set work.save;

drate = &mr_dissat;

srate = &mr_sat;

**** When bias is close to zero SAS may produce a very small negative value

for this term. Here we change the value to zero to keep the square root

from producing a missing value. Since the bias-squared term cannot be

negative we assign it to zero when we get a negative result.

****;

if (mse - sampvar) lt **0** then biassq=**0**;

else biassq= (mse - sampvar);

bias = sqrt(biassq);

keep drate srate mse sampvar bias;

run;

proc append base=work.table data=work.row&row;

run;

%end;

**%mend**;

**%macro** papercall (resp, n, outclear, m, respprof, panel);

%if &outclear=T %then %do;

dm log "clear";

dm "odsresults; clear;";

%end;

ods select none;

proc datasets library=work ;

delete table;

delete test;

run;

quit;

ods select all;

%***wtsim*** (**12345**, **5**, &resp, **0.1**, **0.1**, **3**, &n, **100000**, **1000**, F, **1**, **1**);

%***wtsim*** (**12345**, **5**, &resp, **0.1**, **0.2**, **3**, &n, **100000**, **1000**, F, **2**, **1**);

%***wtsim*** (**12345**, **5**, &resp, **0.1**, **0.3**, **3**, &n, **100000**, **1000**, F, **3**, **1**);

%***wtsim*** (**12345**, **5**, &resp, **0.1**, **0.4**, **3**, &n, **100000**, **1000**, F, **4**, **1**);

%***wtsim*** (**12345**, **5**, &resp, **0.1**, **0.5**, **3**, &n, **100000**, **1000**, F, **5**, **1**);

%***wtsim*** (**12345**, **5**, &resp, **0.1**, **0.6**, **3**, &n, **100000**, **1000**, F, **6**, **1**);

%***wtsim*** (**12345**, **5**, &resp, **0.1**, **0.7**, **3**, &n, **100000**, **1000**, F, **7**, **1**);

%***wtsim*** (**12345**, **5**, &resp, **0.1**, **0.8**, **3**, &n, **100000**, **1000**, F, **8**, **1**);

%***wtsim*** (**12345**, **5**, &resp, **0.1**, **0.9**, **3**, &n, **100000**, **1000**, F, **9**, **1**);

proc print data=test noobs label;

title "Using demographics to detect NMAR";

%if &m=MAR %then %do;

title2 "Data missing at random";

%end; %else %if &m=NMAR %then %do;

title2 "Data not missing at random";

%end;

title3 "Based on n=&n surveys sent out";

title4 "Response Profile: &respprof";

var srate drate race_reject sex_reject conclude_nmar;

run;

proc sgplot data=table;

yaxis min=**0** max=**1**;

series x=drate y=mse / curvelabel ;

series x=drate y=sampvar / curvelabel ;

series x=drate y=bias / curvelabel ;

title "MSE, Bias and Variance by Dissatisfaction Missingness";

%if &m=MAR %then %do;

title2 "Data missing at random";

%end; %else %if &m=NMAR %then %do;

title2 "Data not missing at random";

%end;

title3 "Based on n=&n surveys sent out";

title4 "Response Profile: &respprof";

run;

data panel&panel;

set table;

length row $**20**

col $**8**

;

row="&respprof";

col="n=&n";

panelnum = &panel;

run;

**%mend** papercall;

ods rtf file="C:\Users\pweiss2\Documents\NMAR Sampling Weights\Macro Results.doc";

*** Uniform response ***;

%***papercall*** (**0.2** **0.2** **0.2** **0.2** **0.2**, **800**, F, NMAR, Uniform, **1**);

%***papercall*** (**0.2** **0.2** **0.2** **0.2** **0.2**, **8000**, F, NMAR, Uniform, **2**);

*** Generally Satisfied ***;

%***papercall*** ( **0.1** **0.15** **0.2** **0.3** **0.25**, **800**, F, NMAR, Generally Satisfied, **3**);

%***papercall*** ( **0.1** **0.15** **0.2** **0.3** **0.25**, **8000**, F, NMAR, Generally Satisfied, **4**);

*** Generally Dissatisfied ***;

%***papercall*** ( **0.25** **0.3** **0.2** **0.15** **0.1**, **800**, F, NMAR, Generally Dissatisfied, **5**);

%***papercall*** ( **0.25** **0.3** **0.2** **0.15** **0.1**, **8000**, F, NMAR, Generally Dissatisfied, **6**);

ods rtf close;

**proc** **format**;

value pnf **1**="Uniform, n=800"

**2**="Uniform, n=8000"

**3**="Generally Satisfied, n=800"

**4**="Generally Satisfied, n=8000"

**5**="Generally Dissatisfied, n=800"

**6**="Generally Dissatisfied, n=8000"

;

**run**;

**data** panelplot;

set panel1 - panel6;

format panelnum pnf.;

**run**;

**proc** **sgpanel** data=panelplot;

panelby panelnum / columns=**2** novarname ;

rowaxis values=("Uniform" "Generally Satisfied" "Generally Dissatisfied");

colaxis values=("n=800" "n=8000");

series x=drate y=bias / ; * curvelabel="|Bias|" ;

series x=drate y=mse / ; *curvelabel="MSE";

series x=drate y=sampvar / ; * curvelabel="Var";

title "MSE, Bias and Variance by Dissatisfaction Missingness and Sample Size";

keylegend ;

**run**;
